# Outcomes of En bloc resection followed by reconstruction of giant cell tumor around knee and distal radius. A case series

**DOI:** 10.1016/j.amsu.2019.11.019

**Published:** 2019-12-06

**Authors:** Achmad Fauzi Kamal, Almu Muhamad

**Affiliations:** a Department of Orthopaedic and Traumatology, Cipto Mangunkusumo General Hospital, Faculty of Medicine Universitas Indonesia, Jakarta, Indonesia; b Department of Orthopaedic and Traumatology, Faculty of Medicine Universitas Andalas, Padang, Indonesia

**Keywords:** Giant cell tumor, En bloc resection, Megaprosthesis reconstruction, Arthrodesis, Fibular graft

## Abstract

**Introduction:**

This study is to evaluate the outcomes of En bloc resection and reconstruction in patients with GCT of the bone around the knee and in the distal radius.

**Materials and methods:**

We reviewed 41 cases of GCT of the bone that was treated by En bloc resection around the knee joint and in the distal radius from 2011 to 2018. The local recurrence, metastases, complications and functional score were evaluated for each operation technique.

**Results:**

The average of MSTS score for all group was 78% (excellent). In the knee joint, the megaprostheses group had an excellent MSTS score of78.9% and good 21.1%. The MAMC group had an excellent MSTS score of40.0%, good 50.0% and fair 10.0%. One patient in the megaprostheses group had metastasis to the lung and 1 patient in the knee arthrodesis group has a recurrence. Infection occurred in 2 cases of megaprostheses while only 1 case in MAMC. Both of the groups in knee joint GCT had 1 patient with implant loosening. In the distal radius, FVFG group had an excellent MSTS score 100% and NVFG group had an excellent score 77.7%, good 11.1% and fair 11.1%. One patient in the NVFG group had an infection, 1 patient has implant loosening and another one patient had graft failure. Two patients in the NVFG group had a recurrence. No metastasis was found in both of the group types of surgery in distal radius GCT.

**Conclusion:**

Functional outcome of a patient with GCT of the bone after En bloc resection and reconstruction with the above techniques had comparable results with previous studies.

## Highlights

• Reconstruction after En bloc resection in GCT around knee and distal radius may be done in a variety of techniques.
• Megaprosthesis or modified arthrodesis with MAMC may be used for reconstruction in GCTs around the knee.
• Free or non vascularised fibular graft is a good option for reconstruction after En bloc resection of GCT distal radius.
• Functional outcome of the patients with GCT of the bone after En bloc resection and reconstruction had good results.

## 1 Introduction

Giant cell tumor (GCT) of the bone is a benign aggressive tumor with the ability to metastasize and high recurrence rate after surgery [[1], [2], [3]]. The incidence of GCT of the bone is 5% of all primary bone tumors, with the peak incidence at the age of three and four decades [[1], [2], [3]]. The most common location is the end of along bone such as the distal femur, proximal tibia, distal radius, and proximal humerus. And several cases have been reported in the calcaneus, pelvis and other bones [[2], [3], [4]]. In early stage, the treatment of GCT of the bone can be done by curettage and bone graft, with local recurrence rate of 25–50% [2]. Extended curettage with local chemical adjuvant therapy (including peroxide, liquid nitrogen, fenol, and alcohol) can decrease recurrence rate to 6–25% [2,5]. Wide excision (En bloc) with several technique reconstructions can be done in the late stage of GCT [[2], [3], [4], [5]]. Recently, successful treatment has been reported after targeted therapy with denosumab [2,6].

Post-resection reconstruction may be done in a variety of techniques according to the implant availability and ability of the surgeon. For the GCT around the knee, limb salvage surgery (LSS) may be done with several techniques such as; biological reconstruction with osteoarticular allograft, intercalary allograft, distraction osteogenesis, rotation plasty, knee arthrodesis with autograft (Juvara technique), or endoprothesis (megaprothesis) [[7], [8], [9]].

Previously, En bloc resection followed by arthrodesis (Juvara or modified technique) is the preferred method. Nowdays patients prefer to keep the joint motion and choose reconstruction with megaprosthesis. This technique may achieve a satisfactory functional result but need large and expensive implant [10,11].

**Fig. 1:**
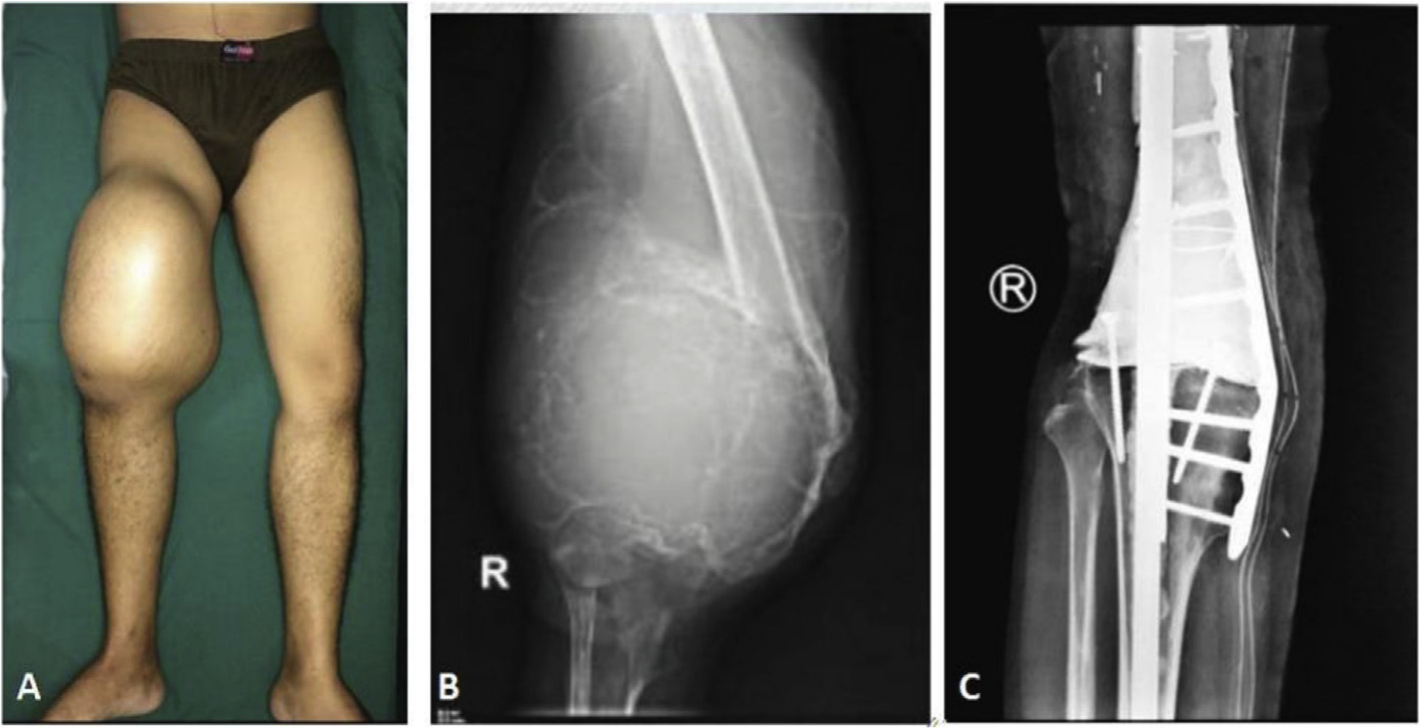
En bloc resection of the GCT at right distal femur and reconstruction with MAMC. A). Clinical picture of huge mass of the GCT at the right distal femur; B).Radiograph of the patient before surgery; C). Postoperative radiograph with MAMC

Many patients with GCT have lately come to our hospital. In this situation, we performed LSS En bloc resection and reconstruction with megaprosthesis or modified arthrodesis with metallic implant plus bone cement (MAMC) in GCTs around the knee (Fig.1) [12]. In the latter technique, after complete resection of the tumor, we filled the defect with intramedullary nailing supported with plate screws and bone cement [12]. We chose the MAMC technique in patients who had much more soft tissue extension without neurovascular bundle involvement or in patients who were not supported by any insurance/government because it is more simple and cheaper than megaprosthesis or Juvara reconstruction For the GCT of the distal radius, free vascularized fibular graft (FVFG) and non vascularized fibular grafts (NVFG) are often used after En bloc resection [13,14]. We also conducted En bloc resection of distal radius GCT and reconstruction fibular graft regarding the previous techniques by other expert surgeons.

This study will evaluate the outcomes of En bloc resection and reconstruction in patients with GCT of the bone around the knee and distal radius.

## 2 Material and methods

The present study is a case series in a single centre institution. We retrospectively reviewed patients with GCT of the bone after wide resection data from musculoskeletal oncology registries, medical records, and follow-up care in an outpatient clinic treated in our hospital, from 2011 to 2018. All lesions were clinically, radiologically, and histologically confirmed as GCT of bone. The patients with GCT of bone other than a distal femur, proximal tibia and distal radius, patients with Campanacci stage 1, and patients who did not complete the profile data and the follow-up of their condition, were excluded. Tumor size was divided into 2 groups (<8 cm and >8 cm).

Part of the subjects with GCT around knee who had had En bloc resection and megaprosthesis reconstruction have published in 2016 [15].

Staging of the tumor was divided into 2 groups (Campanacci stage 2 and 3). Age was classified into groups of decades.Types of reconstruction of GCT around the knee joint were divided into megaprosthesis or MAMC of the knee, whereas the type of reconstruction of distal radius GCT was divided into free vascularized fibular graft (FVFG) and non vascularized fibular graft (NVFG). All surgeries were performed by two orthopaedic oncologic surgeons and supported by one hand and microsurgeon. They are competent in their fields for more than 5 years experience.

We evaluated the complications such as graft failure, infection and loosening of the implant. Local recurrence was detected by physical, radiologic, and histopathology examinations. Lung metastases were confirmed by chest radiograph or computed tomography scan. Functional outcome was assessed at the latest follow-up using the Musculoskeletal Tumor Society (MSTS) score.

The study was reported in line with the PROCESS 2018 criteria [16].

## 3 Results

There were 13 male and 28 female patients aged 17–57 years. The mean age was 35.The most common case was in the 3^rd^ decade. The median follow-up period was 47 (range, 9–88) months.Most of the patients hada mass as a first symptom (58.5%), with a median duration of symptom ofabout 8 months. The most common location of the tumor was the distal femur (39.0%), followed by the proximal tibia (31.7%) and the distal radius (29.3%).Tumors were ≤8 cm in 16 patients and >8 cm in 25 patients. According to the Campanacci staging, tumors were classified as stage 2 (n = 9) and stage 3 (n = 32) (Table 1).

**Table 1:** Characteristics of GCT patients around knee and distal radius in Cipto Mangunkusumo Hospital

Variable	Total (n) (%)
Male	**13 (31.7)**
Female	**28 (68.3)**
Age, years – mean (SD)	**35 (12.93)**
Age group	
11-20-year-old	4 (9.8)
21-30-year-old	16 (39.0)
31-40-year-old	8 (19.5)
>40-year-old	13 (31.7)
ALP, mg/dL – median (min-max)	113 (55–199)
LDH, U/L – median (min-max)	382 (135–745)
Chief complaint, n (%)	
Mass	24 (58.5)
Pain	14 (34.1)
Pain and mass	3 (7.3)
Duration of symptoms, months – median (min-max)	8 (1–72)
Duration of follow-up, months – median (min-max)	47 (9–88)
Tumor size, n(%)	
<8 cms	16 (39.0)
≥8 cms	25 (61.0)
Campanacci staging, n(%)	
2	9 (22.0)
3	32 (78.0)
Tumor location, n(%)	
DR	12 (29.3)
DF	16 (39.0)
PT	13 (31.7)
Biopsy type, n (%)	
Core biopsy	25 (61.0)
FNAB	16 (39.0)
Reconstruction type, n (%)	
Distal radius reconstruction	
FVFG	3 (7.3)
NVFG	9 (22.0)
Around knee joint reconstruction	
Megaprosthesis	19 (46.3)
MAMC	10 (24.4)

ALP, Alkaline Phosphatase; LDH, Lactate Dehydrogenase; FNAB, Fine-Needle-Aspiration Biopsy; DR, Distal Radius; DF, Distal Femur; PT, Proximal Tibia; FVFG, Free Vascularized Fibular Graft; NVFG, Non-vascularized Fibular Graft, MAMC, Modified Arthrodesis of the Knee with Metallic Plus Bone Cement.

**Fig. 2:**
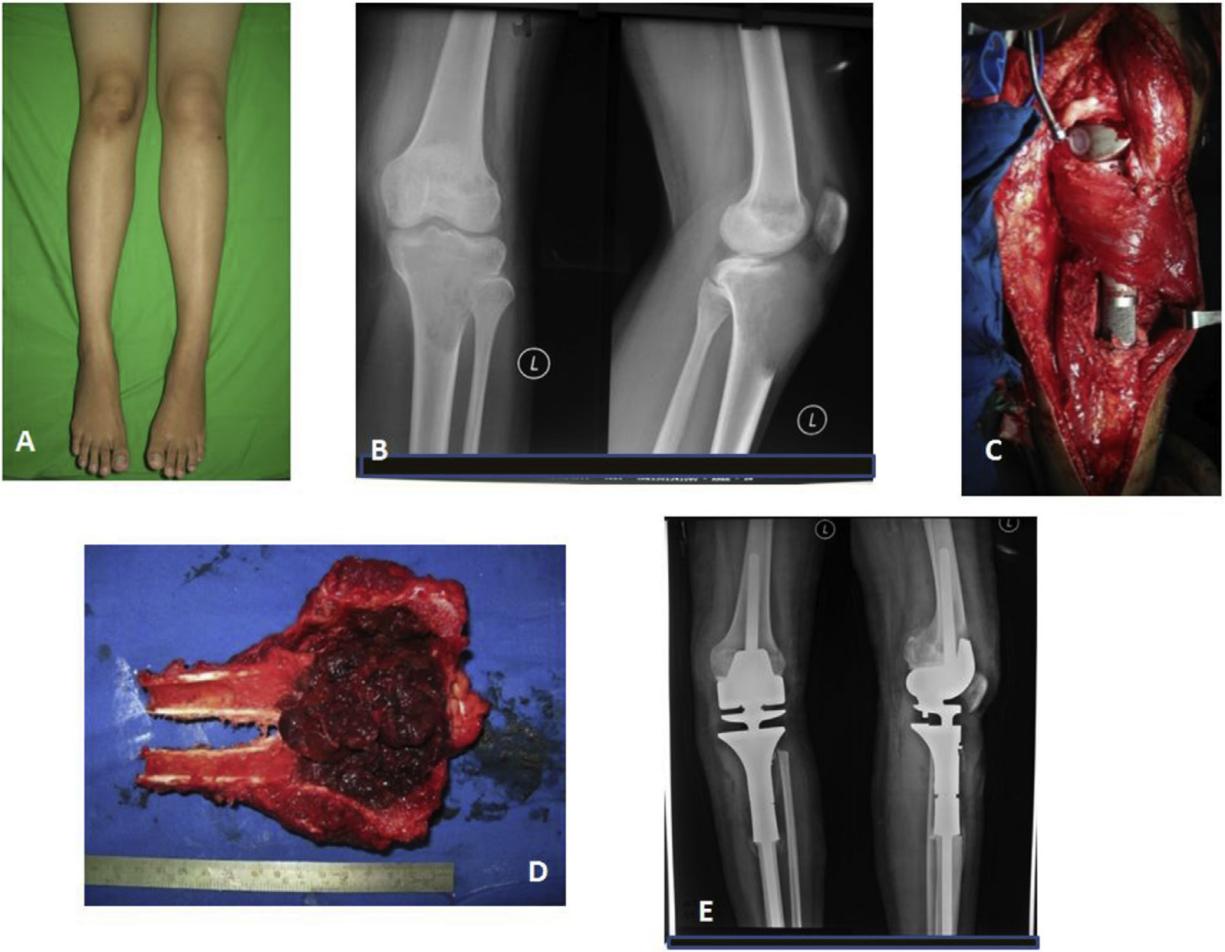
En bloc resection and reconstruction with megaprosthesis of the GCT at proximal tibia. A). Clinical picture of the GCT at the left proximal tibia;B).Radiograph of the patient before surgery; C). Reconstruction of the bone defect with proximal tibia megaprosthesis and medial gatrocnemius flap;D).Gross pathology of the GCT ;E). Postoperative radiograph with megaprosthesis

Of 29 patients who underwent reconstruction inthe knee joint, 19 had a megaprothesis ( Fig. 2) and 10 had knee arthrodesis with MAMC For 12 patients GCT of the distal radius bone, 9 had non-vascularized fibular graft and 3 a free vascularized fibular graft reconstruction.

For all of the patients, the MSTS score was excellent in 70.7% of patients, good in 24.4% of patients, and fair in the remaining patients. The average MSTS score was 78% (excellent).In the knee joint, the megaprosthesis group had an excellent MSTS score of 78.9% and good 21.1%. The MAMC group had an excellent MSTS score 40.0%, good 50.0% and fair 10.0% (Table 2).

**Table 2:** Comparison of the outcomes between megaprosthesis and MAMC reconstructions of GCTs around the knee joint .

	Megaprotheses (n = 19)	MAMC (n = 10)
**MSTS Score**, %, mean (SD)	81.0 (11.64)	74.1 (13.32)
**MSTS Score categories (%)**		
Fair	0	1 (10.0)
Good	4 (21.1)	5 (50.0)
Excellent	15 (78.9)	4 (40.0)
**---**		
Poor-Fair	0	1 (10.0)
Good-Excellent	19 (100.0)	9 (90.0)
**Infection (%)**	2 (10.5)	1 (10.0)
**Implant Loosening (%)**	1 (5.3)	1 (10.0)
**Recurrence (%)**	0	1 (10.0)
**Metastasis (%)**	1 (5.3)	0

Note: Results are shown in mean (SD) for numerical data or frequency (percentage) for proportion.

Infection occurred in 2 cases of megaprosthesis while only 1 case in MAMC. Both of the groups in knee joint GCT had 1 patient with implant loosening. One patient in the knee arthrodesis group had recurrence and 1 patient in the megaprosthesis group had metastasis of the lung.

In the distal radius, FVFG group had an excellent MSTS score 100% and NVFG group had an excellent score 77.7%, a good 11.1% and fair 11.1%. One patient in the NVFG group had an infection, 1 patient had implant loosening and one patient had graft failure. Two patients in the NVFG group had a recurrence (Table 3). No metastasis was found in both of the group types of surgery in the distal radius GCT.

**Table 3:** Comparison of the outcomes between FVFG and NVFG reconstructions of distal radius GCTs .

	FVFG (n = 3)	NVFG (n = 9)
**MSTS Score**, %, mean (SD)	86 (5.13)	75 (12.33)
**MSTS Score categories (%)**		
Fair	0	1 (11.1)
Good	0	1 (11.1)
Excellent	3 (100.0)	7 (77.8)
**---**		
Poor-Fair	0	1 (11.1)
Good-Excellent	3 (100.0)	8 (88.9)
**Infection (%)**	0	1 (11.1)
**Implant Loosening (%)**	0	1 (11.1)
**Graft Failure (%)**	0	1 (11.1)
**Recurrence (%)**	0	2 (22.2)
**Metastasis (%)**	0	0

Note: Results are shown in mean (SD) for numerical data or frequency (percentage) for proportion.

## 4 Discussion

A giant cell tumor of bone is a benign but locally aggressive neoplasm, characterized by a large number of uniformly distributed osteoclast-like giant cells in a bland stroma with epithelioid spindle mononuclear cells. Approximately 60% of giant cell tumors occur adjacent to the knee joint, and the distal radius is the second most common location [17]. We have a similar result with literature in which the most common site in our study is the distal femur.

As locally aggressive lesions which account for about 20% of all benign bone tumors, GCT of the bone occurs in adults between the ages of 20 and 40 years with a slight female dominance [2,3,18,19]. In our study, we also found that GCT had slight female dominance and the most common age is the 3rd decade with GCT being usually present with a short history of pain, mass and functional loss. A mass result from the cortical destruction and tumor progression outside bone [2,4]. In our study, we found mass as a common symptom and most of the patients had a mass with size of more than 8 cm.

GCT is an infrequent and unpredictable lesion. Although several studies have been carried out to predict the behaviour of GCT, there are no definite parameters to determine the prognosis or aggressiveness of this lesion [17]. Different from osteosarcoma that pre-treatment serum LDH and ALP would help to prognosticate patient [20,21], in our study, although most of the patients had elevated LDH and normal ALP, but statistically not significant association with the outcome.

Treatment of GCTs depends on many factors, including the anatomical location, the aggressiveness of the tumor, the functional expectations of the patient, patient's symptoms, the natural history of the tumor and morbidity of treatment [19].The early stage of GCT was treated by intralesional curettage with adjuvant therapies but often associated with a relatively high recurrence rate [[2], [3], [4],^19^]. Saibaba et al. [7] reported recurrent rate after intralesional curettage with adjuvant therapies 24.1% and 5.5% respectively. Some studies mentioned patients with more extensive, biologically aggressive, and/or recurrent GCTs are best treated with enbloc resection.Therefore, in our study, En bloc resection was performed to advanced stage 2 or 3 GCT.

Megaprosthesis is the most common form of reconstruction for stage 2 and 3 GCTs around the knee [22].The reconstruction by megaprosthesis provides immediate stability and allows early mobilisation and weight-bearing. Other benefits are good functional results, especially in the distal femur, excellent cosmesis and patient acceptance, and a relatively low complication rate not affected by on going chemotherapy or radiation [23]. In addition, the surgeons may require megaprosthesis in the setting of recurrence or extensive soft tissue involvement for local control of the tumor [23].

Grimer et al. [24] reported a study of megaprosthesis reconstruction for proximal tibia tumor in 50 patients with the overall MSTS score being 77% (excellent). Another study which was conducted by Sharil et al. [25], demonstrated early functional outcomes of resection and megaprosthesis reconstruction of the distal femur and proximal tibia tumors were a good result. In this present study, the MSTS score of post en-bloc resection and megaprosthesis reconstruction of the GCT around the knee was better than knee arthrodesis with MAMC and a mean MSTS score 81% and 74% respectively for megaprosthesis and MAMC.

In this study, distal radius GCTs had been treated by En bloc resection and reconstructed by free vascularized or non vascularized fibular graft. There are several benefits of the fibular autograft such as: near similar anatomical forms between the distal radius and the proximal fibula, biological reconstruction that is important as the disease occurs in younger patients, whose functional demands are high and who have higher life expectancies, providing continuous remodelling and moulding of the articular surface and limiting chondrolysis [26]. Another benefit of fibular autograft is the good long-term functional outcome and low complication rate [27].Union rate with (free) vascularized bone is more rapid and dependable than with non-vascularized autograft or allograft alone [21].

In the present study, all the patients with free vascularized fibular graft had excellent results, whereas only 77% of patients with non-vascularized fibular graft had excellent functional outcomes. This result is almost similar with other studies by Choo et al. [28] that MSTS score ranged from 70 to 93.3%, by Saini et al. [13] that MSTS score showed 91.4% (76.7–93.3%), and by Jafari et al. [29] that mean modified MSTS score was 75.8 ± 8%.

In our study, we noticed some complications, including infection, implant loosening and graft failure both in a round knee joint and distal radius GCTs. Regarding previous studies, infection was the most common cause of failure and the second most common cause of amputation. The infection influenced the functional outcome, especially as a deep infection could result in a poor functional outcome [25]. Deep infection may acutely occur as a result of contamination during surgery or as a late complication from hematogenous spreading [30], or soft tissue compromise [31].The use of medial gastrocnemius flap in proximal tibia reconstruction may reduce potential infection [32].

In the present study, we had three cases of implant loosening, two cases of knee GCT and another one in distal radius GCT. This result is almost similar to another study by Ng et al. [33] that had 6.4% case of implant loosening, and a study by Heijden et al. [34] that had 8% of cases. Xiu et al. [11] had reported an higher result of implant loosening (31.6%). Adequate soft tissue coverage and proper cementation technique reported can reduce implant loosening rate [35].

We had a broken non-vascularized fibular graft. This result is almost similar to a study by Lenze et al. [36] that reported 9% cases of graft fracture and study by Liu et al. [37] that reported 20% cases of graft fracture. This is a limitation of non vascularized fibular graft. On other hand, the mechanical and structural integrity was preserved in free vascularized fibular graft because this technique allows incorporation between the graft and host bone via primary or secondary bone healing [38,39]. However, non vascularized fibular graft is a simpler, less expensive and a shorter procedure than the use of vascularized graft [37].

Even though we conducted En bloc resection in this present study, local recurrence became an issue in GCT of the bone management [40]. We had three cases of local recurrence, two patients after En bloc resection and non-vascularized fibular graft and a patient after En bloc resection and knee arthrodesis MAMC. Errani et al. [41] reported a recurrencerate of 5% and 12% respectively after En bloc resection of GCTs. And, several authors also reported 5–16% local recurrences in their studies [[40], [41], [42]]. Age at the time of diagnosis is believed as one of predictive factor to local recurrence [40] and may associate with increased bone turn over in young people [43]. Other factors that may influence GCT recurrence are the original stage, surgical margin and biological tumor behaviour [17]. We think that the surgical margin is the most important factor influencing local recurrence.

We had a case of lung metastasis in our study. In some literature, approximately 1–3% of GCTs of bone developed pulmonary metastases [3,17]. There is no clinical or microscopic feature that allows which GCT will metastasis [17]. The risks of pulmonary metastases of GCT will increase in patients who are younger, present with stage 3, develop local recurrence and/or present with axial disease [3,17,44]. In our case metastasis, patients had 3rd decade, with stage 3 proximal tibia GCT without local recurrent.

This study helps us to conclude, in patients with more extensive and biologically aggressive GCTs (stage 2 and 3), en-bloc resection and reconstruction with megaprosthesis or MAMC for a round knee or with massive fibular grafts for distal radius cases are good choices of treatment. Functional outcome of patients with GCT of the bone after En bloc resection and reconstruction with the above techniques had comparable results with another previous study.

## 5 Conclusion

In patients with more extensive and biologically aggressive GCTs (stage 2 and 3), En bloc resection and reconstruction with megaprosthesis or MAMC for a round knee or with massive fibular grafts for distal radius cases are good choices of treatment. Functional outcome of patients with GCT of the bone after En bloc resection and reconstruction with the above techniques had comparable results with another previous study. We still need to evaluate with prospective cohort study.

## Provenance and peer review

Not commissioned, internally reviewed.

## Ethical approval

Faculty of Medicine Universitas Indonesia/Cipto Mangunkusumo General Hospital number KET-536/UN2.F1/ETIK/PPM.00.02/2019.

## Sources of funding

The authors declare that sponsors had no such involvement.

## Author contribution

AFK contributed to performed the operation, data collection, analysis and interpretation, manuscript drafting, revising, and approval for publishing; AM contributed to assist the operation, data collection, analysis and interpretation, manuscript drafting, revising, and approval for publishing.

## Consent

The patient received an explanation of the procedures and possible risks of the surgery, and gave written informed consent.

## Registration number

Research registry no 5076.

## Guarantor

Guarantor in this study is AFK.

## Declaration of competing interest

The authors declare that there is no conflict of interests regarding the publication of this paper.

## References

[1] Kamal A.F., L Simbolon E., Prabowo Y., Hutagalung E.U. (2016). Wide resection versus curettage with adjuvant therapy for giant cell tumour of bone. J. Orthop. Surg..

[2] Anshul S., Agrawal P., Agarwala S., Agarwal M. (2016). Giant cell tumor of bone - an Overview. Arch Bone Jt Surg.

[3] Chakarun C.J., Forrester D.M., Gottsegen C.J., Patel D.B., White E.A., MatcukJr G.R. (2013). Giant cell tumor of bone: review, mimics, and new developments in treatment. RadioGraphics.

[4] Georgiev G.V., Slavchev S., Dimitrova I.N., Landzhov B B. (2014). Giant cell tumor of bone: current review of morphological, clinical, radiological, and therapeutic characteristics. J Clin Exp Invest.

[5] Morii T., Yabe H., Morioka H., Suzuki Y., Anazawa U., Toyama Y. (2018). Curettage and allograft reconstruction for giant cell tumours. J. Orthop. Surg..

[6] Xu S.F., Adams B., Yu X.C., Xu M. (2013). Denosumab and giant cell tumour of bone—a review and future management considerations. Curr. Oncol..

[7] Saibaba B., Chouhan D.K., Kumar V., Dhillon M.S., Rajoli S.R. (2014). Curettage and reconstruction by the sandwich technique for giant cell tumours around the knee. J. Orthop. Surg..

[8] Abed Y.Y., Beltrami G., Campanacci D.A., Innocenti M., Scoccianti G., Capanna R. (2009). Biological reconstruction after resection of bone tumours around the knee. J Bone Jt Surg Br.

[9] Xu X.c., Song, Fu Z.H., Liu X.P. (2010). Long-term outcome of giant cell tumors of bone around the knee treated by en bloc resection of tumor and reconstruction with prosthesis. Orthop. Surg..

[10] Salai M., Nerubay J., Caspi I., Horoszowski H. (1997). Resection arthrodesis of the knee in the treatment of tumours – a long-term follow-up. Int. Orthop..

[11] Rigollino A.V., Fernando T.S., Tanaka M.H., Souza M.M. (2017). Giant cell tumor locally advanced around the knee: treatment and literature review. Rev Bras Ortop.

[12] Kamal A.F., Rubiansyah P. (2019). Clinical outcome of various limb salvage surgeries in osteosarcoma around knee: megaprosthesis, extracorporeal irradiation and resection arthrodesis. Ann Med Surg.

[13] Saini R., Bali K., Bachhal V., Mootha A.K., Dhillon M.S., Gill S.S. (2011). En bloc excision and autogenous fibular reconstruction for aggressive giant cell tumor of distal radius: a report of 12 cases and review of literature. J. Orthop. Surg. Res..

[14] Aithal V.K., Bhaskaranand K. (2003). Reconstruction of the distal radius by fibula following excision of giant cell tumor. Int Orthop (SICOT.

[15] Kamal* A.F., Pitarini A., Prabowo Y. (2018). Megaprosthesis limb salvage surgery: outcome and challenges in treating advanced bone tumour cases in vast archipelago in Indonesia. A case series. Int J Surg Open.

[16] Agha R.A., Borrelli M.R., Vella-Baldacchino M., Thavayogan R., Orgill for the PROCESS Group D.P. (2018). The PROCESS 2018 statement: updating consensus preferred reporting of case series in surgery (PROCESS) guidelines. Int. J. Surg..

[17] Muscolo D.L., Ayerza M.A., Aponte-Tinao L.A. (2001). Giant cell tumours of bone. Curr. Orthop..

[18] Pollock R. (2009). Management of benign bone tumours. Orthop. Traumatol..

[19] Mavrogenis A.F., Igoumenou V.G., Megaloikonomos P.D., Panagopoulos G.N., Papagelopoulos P.J., Soucacos P.N. (2017). Giant cell tumor of bone revisited. Sicot J.

[20] Yahaya S S., Sofian A.M., Mat Saad A.Z., Zulmi W., Nor Azman M.Z., Faisham W.I. (2018). Pre-treatment serum lactate dehydrogenase (LDH) and serum alkaline phosphatase (ALP) as prognostic factors in patients with osteosarcoma. J Cancer Prevention Current Res.

[21] Choong P.F.M., Tumours F.H. Sim (2000). Curr. Orthop..

[22] Faisham W.I., Zulmi W., Halim A.S., Biswal B.M., Mutum S.S., Ezane A.M. (2006). Aggressive giant cell tumour of bone. Singap. Med. J..

[23] Gaston C.L.L., Goulding K., Grimer R.J. (2014). The use of endoprostheses in musculoskeletal oncology. Oper. Tech. Orthop..

[24] Grimer R.J., Carter S.R., Illman R.M., Sneath R.S., Walker P.S., Unwin PS P.S. (1999). Endoprosthetic replacement of the proximal tibia. J Bone Joint Surg [Br].

[25] Sharil A., Nawaz A., Nor Azman M., Zulmi W., Faisham W.I. (2013). Early functional outcome of resection and endoprosthesis replacement for primary tumor around the knee. Mala*ys Orthop*.

[26] Sadek A.F., Abdelfattah A.S., Halim A.S., Faisham W.I., Zulmi W. (2016). Does distal radius reconstruction by free epiphyseal transfer lead to inferior radioulnar dissociation?. Ann Clin Case Reports.

[27] Halim A.S., Chai S.C., Faisham W.I., Azman W.S., Mat Saad A.Z., Zulmi W. (2015). Long-term outcome of free fibula osteocutaneous flap and massive allograft in the reconstruction of long bone defect. J. Plast. Reconstr. Aesthet. Surg..

[28] Choo C.Y., Mat-Saad A.M., Wan-Azman W.S., Zulmi W., Nor-Azman M.Z., Yahaya S. (2018). Functional outcome after treatment of aggressive tumours in the distal radius: Comparison between reconstruction using proximal fibular graft and wrist fusion. Malays Orthop J.

[29] Jafari D., Shariatzadeh H., Okhovatpour M.A., Razavipour M., Saf F. (2017). Giant cell tumor of distal radius: en bloc resection and partial wrist arthrodesis using non-vascularized fibular autograft. Shafa Orthop J.

[30] Seeger L.L., Farooki L.L.S., Yao L., Kabo J.M., Eckardt J.J. (1998). Custom endoprostheses for limb salvage: a historical perspective and imaging evaluation. AJR Am. J. Roentgenol..

[31] Morii T T., Morioka H., Ueda T., Araki N., Hashimoto N., Kawai A. (2013). Deep infection in tumor endoprosthesis around the knee: a multi-institutional study by the Japanese musculoskeletal oncology group. BMC Muscoskelet. Disord..

[32] Cannon S.R. (1997). Massive prostheses for malignant bone tumours of the limbs. J. Bone Jt. Surg..

[33] Ng E.S., Saw A., Sengupta S., Nazarina A.R., Path M. (2002). Giant cell tumour of bone with late presentation: review of treatment and outcome. J. Orthop. Surg..

[34] van der Heijden L., Dijkstra P.D., Campanacci D.A., Gibbons C.L., van de Sande M.A. (1999). Giant cell tumor with pathologic fracture: should we curette or resect?. Clin. Orthop. Relat. Res..

[35] Gkavardina A., Tsagozis P. (2014). The use of megaprostheses for reconstruction of large skeletal defects in the extremities: a critical review. Open Orthop. J..

[36] Lenze U., Kasal S., Hefti F., Krieg A.H. (2017). Non-vascularised fibula grafts for reconstruction of segmental and hemicortical bone defects following meta-/diaphyseal tumour resection at the extremities. BMC Muscoskelet. Disord..

[37] Liu S., Tao S., Tan J., Hu X., Liu H., Li Z. (2018). Long-term follow-up of fibular graft for the reconstruction of bone defects. Medicine (Baltim.).

[38] El-Sayed M., El-Hadidi M., El-Ad W. (2007). Free non-vascularised fibular graft for treatment of post-traumatic bone defects. Acta Orthop. Belg..

[39] Bae D.S., Waters P.M. (2008). Free Vascularized Fibula Grafting: Principles,techniques, and Applications in Pediatric Orthopaedics.

[40] Klenke F.M., Wenger D.E., Inwards C.Y., Rose P.S., Sim F.H. (2011). Giant cell tumor of bone risk factors for recurrence. Clin. Orthop. Relat. Res..

[41] Errani C., Ruggieri P., Asenzio M.A., Toscano A., Colangeli S., Rimondi E. (2010). Giant cell tumor of the extremity: a review of 349 cases from a single institution. Cancer Treat Rev..

[42] Kim Y., Nizami S., Goto H., Lee F.Y. (2012). Modern interpretation of giant cell tumor of bone: predominantly osteoclastogenic stromal tumor. Clin. Orthop. Surg..

[43] Demontiero O., Vidal C., Duque G. (2012). Aging and bone loss: new insights for the clinician. Ther Adv Musculoskelet Dis.

[44] Chan C.M., Adler Z., Reith J.D., Gibbs C.P. (2015). Risk factors for pulmonary metastases from giant cell tumour of bone. J Bone Joint Surg Am.

