# Supplementary material for: Outcomes of En bloc resection followed by reconstruction of giant cell tumor around knee and distal radius. A case series
Source: Ann Med Surg (Lond). 2019 Dec 6;49:61–6. doi: 10.1016/j.amsu.2019.11.019 (PMC6926124; doi:10.1016/j.amsu.2019.11.019)
Supplement: Multimedia component 2 [file mmc2.docx]

| PROCESS CHECKLIST | | | |
| --- | --- | --- | --- |
| Section | **Item** | **Checklist Description** | **Page Number** |
| Title | 1 | The words “case series” and the area of focus should appear in the title (e.g. disease, exposure/intervention or outcome). | 1 |
| Abstract | 2a | **Introduction**  What is the unifying theme of the case series. : **Outcomes of En bloc resection followed by reconstruction of giant cell tumor** | 1  1  1  1 |
|  | 2b | **Methods**  Describe what was done, how and when was it done and by whom. |  |
|  | 2c | **Results**  What was found. |  |
|  | 2d | **Conclusion**  What have we learned and what does it mean |  |
| Introduction | 3 | Explain the scientific background and rationale for the case series. What is the unifying theme - common disease, exposure, intervention and outcome, etc. Why is this study needed? | 2 |
| Methods | 4a | **Registration and ethics**  State the research registry number in accordance with the declaration of Helsinki - "Every research study involving human subjects must be registered in a publicly accessible databasebefore recruitment of the first subject" (this can be obtained from; ResearchRegistry.com or ClinicalTrials.gov or ISRCTN). Even retrospective studies should be registered prior to submission. State whether ethical approval was needed and if so, what the relevant judgement reference from the IRB or local ethics committee was? If ethical approval was not needed, state why. | 3 |
|  | 4b | **Study design**  State the study is a case series and whether prospective or retrospective in design, whether single or multi-centre and whether cases are consecutive or non-consecutive. | 3 |
|  | 4c | **Setting**  Describe the setting(s)and nature of the institution in which the patient was managed; academic, community or private practice setting? Location(s), and relevant dates, including periods of recruitment, exposure, follow-up, and data collection | 3 |
|  | 4d | **Participants**  Describe the relevant characteristics of the participants (comorbidities, tumour staging, smoking status, etc). State any eligibility (inclusion/exclusion) criteria and the sources and methods of selection of participants. Describe length and methods of follow-up.  We retrospectively reviewed patients with GCT of the bone after wide resection data from musculoskeletal oncology registries, medical records, and follow-up care in an outpatient clinic treated in Orthopaedic and Traumatology Department, Cipto Mangunkusumo Hospital, Jakarta from 2011 to 2018. All lesions were clinically, radiologically, and histologically confirmed as GCT of bone. The patients with GCT of bone other than a distal femur, proximal tibia and distal radius, patients with Campanacci stage 1, and patients who did not complete the profile data and the follow-up of their condition, were excluded.  Part of the subjects with GCT around knee who had had En bloc resection and megaprosthesis reconstruction have published in 2016 [15].  Staging of the tumor was divided into 2 groups (Campanacci stage 2 and 3). Age was classified into groups of decades.Types of reconstruction of GCT around the knee joint were divided into megaprosthesis or MAMC of the knee, whereas the type of reconstruction of distal radius GCT was divided into free vascularized fibular graft (FVFG) and non vascularized fibular graft (NVFG). All surgeries were performed by two orthopaedic oncologic surgeons and supported by one hand and microsurgeon. They are competent in their fields for more than 5 years experience. | 3 |
|  | 4e | **Pre-intervention considerations**  e.g. Patient optimisation: measures taken prior to surgery or other intervention e.g. treating hypothermia/hypovolaemia/hypotension in burns patients, ICU care for sepsis, dealing with anticoagulation/other medications and so on.  Many patients with GCT have lately come to our hospital. In this situation, we performed LSS En bloc resection and reconstruction with megaprosthesis or modified arthrodesis with metallic implant plus bone cement (MAMC) in GCTs around the knee [12]. In the latter technique, after complete resection of the tumor, we filled the defect with intramedullary nailing supported with plate screws and bone cement [12]. We chose the MAMC technique in patients who had much more soft tissue extension without neurovascular bundle involvement or in patients who were not supported by any insurance/government because it is more simple and cheaper than megaprosthesis or Juvara reconstruction (Figure). | 2 |
|  | 4f | **Types of intervention(s) deployed**  To include reasoning behind treatment offered (pharmacological, surgical, physiotherapy, psychological, preventive) and concurrent treatments (antibiotics, analgesia, anti-emetics, nil by mouth, VTE prophylaxis, etc). Medical devices should have manufacturer and model specifically mentioned. | 2-3 |
|  | 4g | **Peri-intervention considerations**  Administration of intervention (what, where, when and how was it done, including details for surgery; anaesthesia, patient position, use of tourniquet and other relevant equipment, preparation used, sutures, devices, surgical stage (1 or 2 stage, etc) and operative time. Pharmacological therapies should include formulation, dosage, strength, route and duration). Authors are encouraged to use figures, diagrams, photos, video and other multimedia to explain their intervention. | 2-3 |
|  | 4h | **Who performed the procedure(s)**  Operator experience (position on the learning curve for the technique if established, specialisation and prior relevant training). All surgeries were performed by two orthopaedic oncologic surgeons and supported by one hand and microsurgeon. They are competent in their fields for more than 5 years experience. | 3 |
|  | 4i | **Quality control**  What measures were taken to reduce inter or intra-operator variation. What measures were taken to ensure quality and consistency in the delivery of the intervention e.g. independent observers, lymph node counts, etc  We had a clinicopathological conference and pre-postoperative report every weak. | 3 |
|  | 4j | **Post-intervention considerations**  e.g. post-operative instructions and place of care. Important follow-up measures - diagnostic and other test results. Future surveillance requirements - e.g. imaging surveillance of endovascular aneurysm repair (EVAR) or clinical exam/ultrasound of regional lymph nodes for skin cancer. | 3 |
| Results | 5a | **Participants**  Report numbers involved and their characteristics(co-morbidities, tumour staging, smoking status, etc). |  |
|  | 5b | **Changes**  Any changes in the interventions during the course of the case series (how has it evolved, been altered or tinkered with, what learning occurred, etc) together with rationale and a diagram if appropriate. Degree of novelty for a surgical technique/device should be mentioned and a comment on learning curves should be made for new techniques/devices. | 3-4 |
|  | 5c | **Outcomes and follow-up**  Clinician assessed and patient-reported outcomes (when appropriate) should be stated with inclusion of the time periods at which assessed. Relevant photographs/radiological images should be provided e.g. 12-month follow-up. | 3-4 |
|  | 5d | **Intervention adherence/compliance and tolerability**  How was this assessed. Describe loss to follow-up (express as a percentage and a fraction) and any explanations for it. |  |
|  | 5e | **Complications and adverse or unanticipated events**  Described in detail and ideally categorised in accordance with the Clavien-Dindo Classification. How they were prevented, mitigated, diagnosed and managed. Blood loss, wound complications, re-exploration/revision surgery, 30-day post-op and long-term morbidity/mortality may need to be specified. | 4 |
| Discussion | 6a | **Summarise key results** | 4-6 |
|  | 6b | **Discussion of relevance**  Relevant literature, implications for clinical practice guidelines, how have the indications for a new technique/device been refined and how do outcomes compare with established therapies and the prevailing gold standard should one exist and any relevant hypothesis generation. | 4-7 |
|  | 6c | **Strengths and limitations of the study** | 7 |
|  | 6d | **The rationale for any conclusions?** | 7 |
| Conclusions | 7a | **State the key conclusions from the study** | 8 |
|  | 7b | **State what needs to be done next, further research with what study design.** | 8 |
| Additional Information | 8a | **State any conflicts of interest** | 8 |
|  | 8b | **State any sources of funding** | 8 |
